# Intravenous Catheter-Associated Candidemia due to *Candida* *membranaefaciens*: The First Iranian Case

**Published:** 2015-04-03

**Authors:** Seyed Reza Aghili, Tahereh Shokohi, Mohammad Ali Boroumand, Shirinsadat Hashemi Fesharaki, Bahar Salmanian

**Affiliations:** 1 *Department of Medical Mycology and Parasitology, and Invasive Fungi Research Center (IFRC), Faculty of Medicine, Mazandaran University of Medical Sciences, Sari, Iran. *; 2 *Tehran Heart Center, Tehran University of Medical Sciences, Tehran, Iran. *; 3 *Student Research Committee, Faculty of Medicine, Mazandaran University of Medical Sciences, Sari, Iran. *; 4 *Department of Sciences, Seddigheh Tahereh Branch, Farhangian University, Sari, Iran*.

**Keywords:** Cross infection, Catheter-related infections, Candidemia, Coronary artery bypass, Polymerase chain reaction

## Abstract

The incidence of candidemia due to the uncommon non-albicans Candida species appears to be increasing, and certain species such as Candida (C.) membranaefaciens have been reported in some clinical researches. Vascular catheters are considered the likely culprit for the sudden emergence of hospital-acquired candidemia. The identification of C. membranaefaciens can be problematic in clinical practice owing to its phenotypic resemblance to C. guilliermondii. We report the first case of C. membranaefaciens in Iran, which occurred in a 70-year-old woman, who had coronary artery bypass grafting (CABG). We isolated germ-tube negative yeast from both blood culture and central venous catheter (CVC) tip culture on brain-heart infusion agar, Sabouraud dextrose agar plates, and biphasic brain-heart infusion media bottle; it developed smooth, pink colonies on CHROMagar Candida. By using the polymerase chain reaction and sequencing of theinternal transcribed spacer region of rDNA, we identified C. membranaefaciens. After the removal of the CVC and initiation of Fluconazole treatment, the patient's condition gradually improved and she was discharged from the hospital. The early detection of organisms in the catheter, removal of the catheter, and treatment with anti-fungal antibiotics have an important role in controlling disease and preventing septicemia after CABG. As C. membranaefaciens is an opportunistic Candida species, both clinicians and microbiologists should be aware of the factors that confer fast diagnosis and appropriate treatment.

## Introduction

*Candida* (C.) species can be the cause of bloodstream infection in hospitalized and immunocompromised patients.^1^ Known risk factors for candidemia include the use of central-venous catheterization, total parenteral nutrition, previous multiple antibiotics, previous steroid therapy, previous abdominal surgery, and an immunocompromised status.^2^^, ^^3^ The role of catheters in the development of candidemia is obvious. Numerous studies have identified the intravascular catheter, and primarily the central venous catheter (CVC), as a risk factor for the development^4^^-^^6^ and persistence^7^ of *Candida* bloodstream infections. Catheters can be considered as the nidus for the formation of a septic thrombus, but they can also become infected via several routes.^8^ Generally, any type of catheter may become contaminated as a result of the use of the hub and the migration of bacteria or fungi along the internal surface of the catheter. Much less commonly, catheters may become infected as a result of hematogenous seeding or contamination of the transfusion.^9^ It seems that *Candida* species adhere to a catheter with their surface receptors, which allow adherence to the thrombin biofilm that forms on the catheter.^10^

Hydrophobic interactions between the *Candida* surface proteins and the plastic itself may also promote adherence.^11^^, ^^12^

Most of these infections are caused by *Candida albicans*; however, in recent years, nearly 50% of all candidemia cases have been caused by non-albicans species.^13^^, ^^14^ *C. membranaefaciens* has been frequently isolated as *Pichia membranaefaciens* (the teleomorph of *C. membranaefaciens*) from many habitats and substrates, including lake water, bodies of plants, and animals such as scaled insects and termites. C. membranaefaciens has been identified in cases of fungemia and other fungal diseases in humans and animals.^15^^-^^19^ Some studies have shown a phylogenetic relationship between this group and C. guilliermondii. Also, *C. melibiosi* is an obsolete synonym of C. membranaefaciens and was previously known as C. majoricensis.^20^ Since 1988, when the first case occurred, the incidence of C. guilliermondii fungemia has been 2 per 1000 admissions.^21^ The incidence of candidemia due to this uncommon *Candida* species seems to be increasing.The identification of these species can be problematic in routine clinical practice because of their phenotypic resemblance. 

Recent advances in molecular biological methods have drastically changed our understanding of the diversity and evolution of fungi. The progress in DNA sequence-based techniques notably enables us not only to overcome the potential flaws of the traditional mycological techniques but also to evaluate fungal richness more efficiently and reliably.^22^ Indeed, DNA barcoding is now obtained with universal genetic markers and high-quality sequence databases and is, thus, more reliable.^23^

The internal transcribed spacer (ITS) region of the nuclear ribosomal repeat unit is the most popular locus for species identification and sub-generic phylogenetic inference in sequence-based mycological research for yeast fungi, especially *Candida* species. Some researchers have reported that the identification of medically important yeasts by ITS sequencing, especially using the ITS2 region, is reliable and can be used as an accurate alternative and confirmative to the conventional identification methods.^24^

The Basic Local Alignment Search Tool (BLAST) finds regions of local similarity between sequences. Using only the top BLAST hit to classify an unknown sequence is known to be potentially misleading.^25^^, ^^26^ Despite this, BLAST analyses are often used as the basis for species identification to complete or confirm the other methods.

There have been no prior case reports of *C. membranaefaciens* candidemia in Iran; accordingly, the prevalence of this species among Iranians remains unclear. In this study, we report the first case of *C. membranaefaciens* intravenous catheter-associated candidemia in Iran. Inidentification, the case was morphologically and physiologically distinguished as *C. guilliermondii*; however, it was determined as C. membranaefaciens in the BLAST analysis of the ITS region.

## Case Report

A 70-year-old woman was admitted to Tehran Heart Center with a complaint of dyspepsia, unstable angina, and resting retro-sternal chest pain of 30 minutes' duration in August 2012 for further evaluation. She had diabetes for 10 years and complained of several months of intermittent, low-grade fever and night sweats. In initial examinations, her blood pressure was 190/150 mmHg and her thyroid glands were slightly larger than normal. The patient's medical record showed the existence of a severe allergy to color materials and cholecystectomy surgery 4 years before. After angiography, it was found that the patient had atherosclerosis with the stenosis of 3 coronary vessels. On the 5^th^ day after admission, she underwent coronary artery bypass grafting (CABG) with the grafting of the 3 vessels, without any intraoperative complications. After the induction of anesthesia and tracheal intubation, a CVC was placed through the right subclavian vein. On the 8^th^ day after admission (3^rd^ postoperative day), she developed fever for 3 days (37.7 ^°^C, 38 ^°^C, and 38.5 ^°^C, respectively). No bacteria or fungi were seen on the Gram stain examination of blood culture. Intravenous Clindamycin (500 mg IV twice daily) and Ceftriaxone (one g IV a single daily dose) were started empirically. Blood samples were collected and inoculated into biphasic standard blood culture bottles (BBHI) containing brain-heart infusion agar (BHIA)/brain-heart infusion broth (BHIB) (Padtan Teb Co. Tehran, Iran) and were incubated at 37 ^°^C for up to 7 days. On the 4^th^ day of incubation, the blood culture bottles became positive, and subculture yielded Gram-negative, oxidase-negative bacteria. The microorganism was identified as Citrobacter freundii. Based on the antibacterial susceptibility test, Clindamycinwas discontinued and Ceftriaxone (2 g IV once daily) was replaced for another 5 days. After 4 days, the patient became afebrile. On the 8^th^ postoperative day, the last CVC was removed and sent to the pathology laboratory. The blood sample was cultured into standard blood culture bottles and the BBHI media bottle, and the catheter tip was rolled on BHI agar, blood agar, and MacConkey agar plates. The suspension internal lumen of the catheter was cultured in BBHI media as well. Yeast growth was observed on both BHI agar and BBHI media after 72 hr of incubation at 37 ^°^C, although the patient had no fever. On the 5^th^ day of the treatment with Fluconazole (800 mg on the first day, followed by 400 mg once daily for 4 days), the blood cultures were negative. The treatment was continued for another 2 weeks, after which the blood cultures became negative. For further identification of the yeast species, the isolates were referred to the Medical Mycology Department, Faculty of Medicine, Mazandaran University of Medical Sciences, Sari, Iran. 

**Figure 1:**
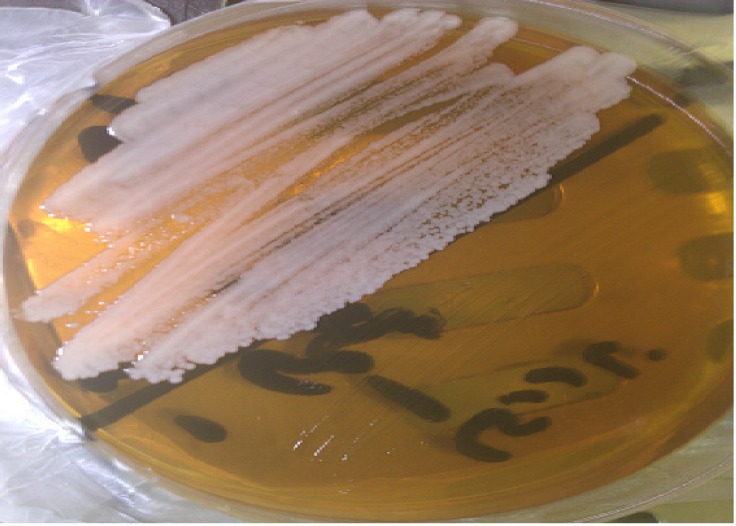
*Candida* *membranaefaciens* on CHROMagar *Candida* plate, incubated at 37 ^°^C for 48 hours

Identification was made on the basis of a combination of various phenotypic methods and confirmed by sequencing the ITS region of the polymerase chain reaction (PCR) product. The isolates did not produce germ tubes in humanpooled serum in 3 hr at 37 ^°^C. In CMA + TW80 medium (Merck, Germany), it produced hyphae or pseudohyphae but no chlamydospores. In addition, the result was negative in the hydrolysis of urea. The isolates were cultured on CHROMagar *Candida* medium (bioMérieux, France) at 37 ^°^C for 48 hr. The resulting colonies were pink in color (Figure 1). 

Genomic DNA was extracted, using the method of glass bead phenol chloroform disruption,^27^ and PCR was amplified by using a modified Shokohi method.^28^ The ITS sequencing was used for the identification of the species. The ITS region of the ribosomal DNA regions were amplificated by universal fungal primers, ITS1 and ITS4, (TCCGTAGGTGAACCTGCGG/GCATATCAATAAGCGGAGGA) (MWG-Biotech AG, Germany). Briefly, to amplify the ITS domains, PCR amplification was performed in a final volume of 25 µl. The reaction consisted of 2.5 µl of template DNA, 0.5 µl of each primer at 12.5 µl Master Mix (Takapouzist Biotech, Iran), and 9.5 µl of sterile deionized water. The amplification parameters consisted of 35 cycles of denaturation at 95 ^°^C for 45 sec, primer annealing at 58 ^°^C for 45 sec, and extension at 72 ^°^C for one min. In the first cycle, the denaturation step was 95 ^°^C for 5 min; and in the final cycle, the final extension step was 72^ °^C for 6 min. The amplified products were visualized by 1% Agarose gel electrophoresis in TBE buffer (20 mmol/L EDTA, 10 mmol Tris boric pH 8). The gel was stained with ethidium bromide (0.5 µg/ml) and photographed by ultraviolet photography. 

Single bands were observed at 600 bp. The PCR product was sent for automated sequencing (Bioneer, Korea). A sequence-similarity search was done using Nucleotide Sequence Databases in National Center for Biotechnology Information/ Basic Local Alignment Search Tool in NCBI BLAST (www.ncbi.nlm.nih.gov/BLAST/). Both isolates were identified as *C. membranaefaciens*, and the sequence was submitted to the GenBank® Nucleotide Sequence Database. The amplification length of the ITS region, substrate, and ITS accession number are listed in Table 1.

**Table 1:** *Candida membranaefaciens* isolated from samples of the patient

Substrate	Length of amplified fragment of the ITS region	Strain	ITS accession No.
Blood sample	587 bp	*Candida membranaefaciens* MUCL. 30392	EU343844.1
Catheter tip	594 bp	*Candida* membranaefaciens MUCL. 30392	EU343844.1

ITS, Internal transcribed spacer

## Discussion

*Candida* is one of the leading causes of bloodstream infections. Indeed, it carries high morbidity and mortality rates and is becoming increasingly prevalent in the world. Of all episodes of candidemia, an estimated 33-55% occurs in the intensive care unit (ICU) environment and is associated with mortality rates ranging from 5% to 71%.^14^ There are a number of conditions predisposing patients to candidemia, including the use of intravascular catheters, use of urinary catheters, prior abdominal surgery, broad-spectrum antibiotics and corticosteroids, malignancy, parenteral nutrition, and prolonged ICU stays.^14^ Among these risk factors, the use of the CVC is the most likely factor for nosocomial candidemia.^29^^, ^^30^ Among the *Candida* species identified up to now, C. albicans has been considered as the first common cause of candidemia; nonetheless, in recent years, the frequency of the episodes of candidemia caused by *Candida* species other than C. albicans has risen.^31^ Although *C. membranaefaciens* is rarely found in the skin microflora of normal healthy individuals and mostly is isolated from water, plants, and scale insects and termites, it has recently been described as an opportunistic yeast associated with blood infection in human patients.^15^^-^^19^ Phenotypically and biochemically, C. membranaefaciens and *C. guilliermondii* have many similarities, including their microscopic morphology, formation of pink to purple colonies on CHROMagar *Candida* medium, and inability to form germ tubes in serum. The majority of the studies in the existing literature have most probably failed to distinguish these two species correctly. Despite the claims that tests based on assimilation and fermentation substratescan differentiate between these two species, these characteristics are not definitive, and the identification of *C. membranaefaciens* in routine laboratory testing remains a problem. C. guilliermondii can be isolated from the human skin and is a well-documented agent of invasive candidiasis.^21^ Masala et al. reported 5 patients with fungemia caused by C. guilliermondii in a surgical unit of their hospital.^32^ *C. membranaefaciens* as well as the other species of *Candida* can adhere and colonize to the CVC line. 

Appropriate patient management is still controversial. Some researchers have documented resistance to Fluconazole for *C. membranaefaciens*.^15^^, ^^19^ Nevertheless, uncommon *Candida* species such as *C. guilliermondii* and *C. membranaefaciens* have susceptible dose-dependent minimum inhibitory concentration (MIC) values for Fluconazole.^33^ In this study, our patient, who had catheter-associated fungemia due to C. membranaefaciens, was treated successfully with catheter removal and Fluconazole.

Tests based on the same phenotypic characteristics such as the inability to grow at 45 ^°^C, non-production of germ tubes in human pooled serum in 3 hr at 37 ^°^C, production of hyphae or pseudohyphae, non-production of chlamydospores in Corn Meal agar with Tween 80 (CMA + TW80) medium, non-hydrolysis of urea, and formation of pink colonies on CHROMagar *Candida* medium cannot differentiate between C. membranaefaciens and *C. guilliermondii*.

Molecular techniques employing PCR have also increased the accuracy of identifying uncommon *Candida* species. The ITS region of rDNA gene sequencing is a useful and definitive method for most *Candida* species, but it requires a specialized instrument to analyze the product. However, PCR using a specific primer can serve as an alternative that is easy to perform and is an accurate diagnostic tool for the identification of *C. membranaefaciens*. In this report, we described the case of *C. membranaefaciens* intravenous catheter-associated candidemia in a 70-year-old woman, who had CABG. This is the first report of candidemia due to *C. membranaefaciens* as confirmed by molecular methods in Iran. During the diagnostic process, the *C. membranaefaciens* infection should be distinguished from infections caused by other *Candida* species, particularly *C. guilliermondii*.

## Conclusion

*Candida* species attach to the vascular catheter material; consequently, an early detection of organisms in the pus or aspiration of the thrombus in the catheter, removal of the catheter, and treatment with antibiotics (anti-bacterial and anti-fungal) play an important role in controlling the disease and preventing septicemia after CABG. As C. membranaefaciens is an opportunist organism, both clinicians and microbiologists should be aware of the factors than can confer speedy diagnosis and appropriate treatment.Research has shown that it is necessary to determine the catheter sample and blood in cardiac patients who have fever and do not respond to prophylactic antibacterial antibiotics; in addition, initiating antifungal prophylaxis in high-risk CABG patients constitutes proper treatment.

## Notes:

***This paper should be cited as: ***Aghili SR, Shokohi T, Boroumand MA, Hashemi Fesharaki SH, Salmanian B. Intravenous Catheter-Associated Candidemia due to *Candida Membranaefaciens*: The First Iranian Case. J Teh Univ Heart Ctr 2015;10(2):101-105.
